# Networks as tools for defining emergent properties of microbiomes and their stability

**DOI:** 10.1186/s40168-024-01868-z

**Published:** 2024-09-28

**Authors:** Kacie T. Kajihara, Nicole A. Hynson

**Affiliations:** Pacific Biosciences Research Center, University of Hawai’i at Mānoa, Honolulu, HI 96822 USA

**Keywords:** Co-occurrence networks, Microbiome, Stability, Fungi, Bacteria, Data science, Multi-omics

## Abstract

The potential promise of the microbiome to ameliorate a wide range of societal and ecological challenges, from disease prevention and treatment to the restoration of entire ecosystems, hinges not only on microbiome engineering but also on the stability of beneficial microbiomes. Yet the properties of microbiome stability remain elusive and challenging to discern due to the complexity of interactions and often intractable diversity within these communities of bacteria, archaea, fungi, and other microeukaryotes. Networks are powerful tools for the study of complex microbiomes, with the potential to elucidate structural patterns of stable communities and generate testable hypotheses for experimental validation. However, the implementation of these analyses introduces a cascade of dichotomies and decision trees due to the lack of consensus on best practices. Here, we provide a road map for network-based microbiome studies with an emphasis on discerning properties of stability. We identify important considerations for data preparation, network construction, and interpretation of network properties. We also highlight remaining limitations and outstanding needs for this field. This review also serves to clarify the varying schools of thought on the application of network theory for microbiome studies and to identify practices that enhance the reproducibility and validity of future work.

Video Abstract

**Supplementary Information:**

The online version contains supplementary material available at 10.1186/s40168-024-01868-z.

## Introduction

With the advancement of new technologies applied to the field of biology, a new perspective has emerged of our place in the biosphere. We now recognize that microbes underpin the function of all sectors of the planet, and we, in fact, live in a microbial world [1]. From mountains to oceans and from the bottom to the top of all food webs, ecosystem and organismal health rely upon the microbiome (communities of bacteria, archaea, fungi, and other microeukaryotes). Thus, harnessing the beneficial properties of microbiomes that support the health of hosts and habitats is an increasingly important pursuit as we seek more personalized medicine, as global change continues to disrupt ecosystems, and as access to nutritious food and clean water is challenging even among some of the wealthiest nations. However, fundamental to the success of efforts such as microbiome engineering is understanding microbiome stability, the ability of a microbiome to resist or recover from disturbances. Stable microbiomes are commonly assessed via DNA sequencing to identify compositional or functional traits that persist in the face of disturbances, ranging from oral antibiotics in humans affecting the gut microbiome [2] to the ability of corals to withstand thermal stress [3]. However, these methods do not necessarily consider the diversity and complexity of interactions that characterize microbial life and potentially foster community stability [4]. To address this, co-occurrence networks have emerged by way of graph theory as a way to model communities in the context of their potential interactions [5]. Co-occurrence analyses have long been used in community ecology to study the architecture of food webs, and many of the concepts used to study microbiome co-occurrence networks originate from these works [6–9]. Given that no microbe acts in isolation, network stability research offers pertinent insights to guide future research for microbiome design and engineering [10, 11].

The co-occurrence networks discussed here are composed of nodes and edges, where nodes can represent microbial taxa, genes, metabolites, or other compositional properties of the microbiome and edges indicate statistically significant relationships between them. In brief, networks are constructed via pairwise comparisons to determine whether there is a significant likelihood that the given microbes tend to co-occur or trend towards mutual exclusion. Edges can be classified as either positive or negative and by the strength of the predicted association. Negative edges potentially represent relationships such as competition or predation, and positive edges could indicate mutualism or commensalism, and experimental co-cultivation has previously supported the fidelity of some edges inferred in silico [12–14]. Each resulting network represents a snapshot of the microbiome that can be used to characterize potential interaction patterns and to predict stability and be comparable to other networks similarly constructed [15]. However, the path to implementing network construction and downstream analyses like stability is littered with considerations, and few standards currently exist among the research community. Beyond this, the network topological metrics used as indicators of stability (e.g., degree, connectivity, or clustering coefficient) often co-correlate [16] or are associated with multiple plausible but conflicting interpretations, further challenging the interpretation of these analyses.

**Fig. 1:**
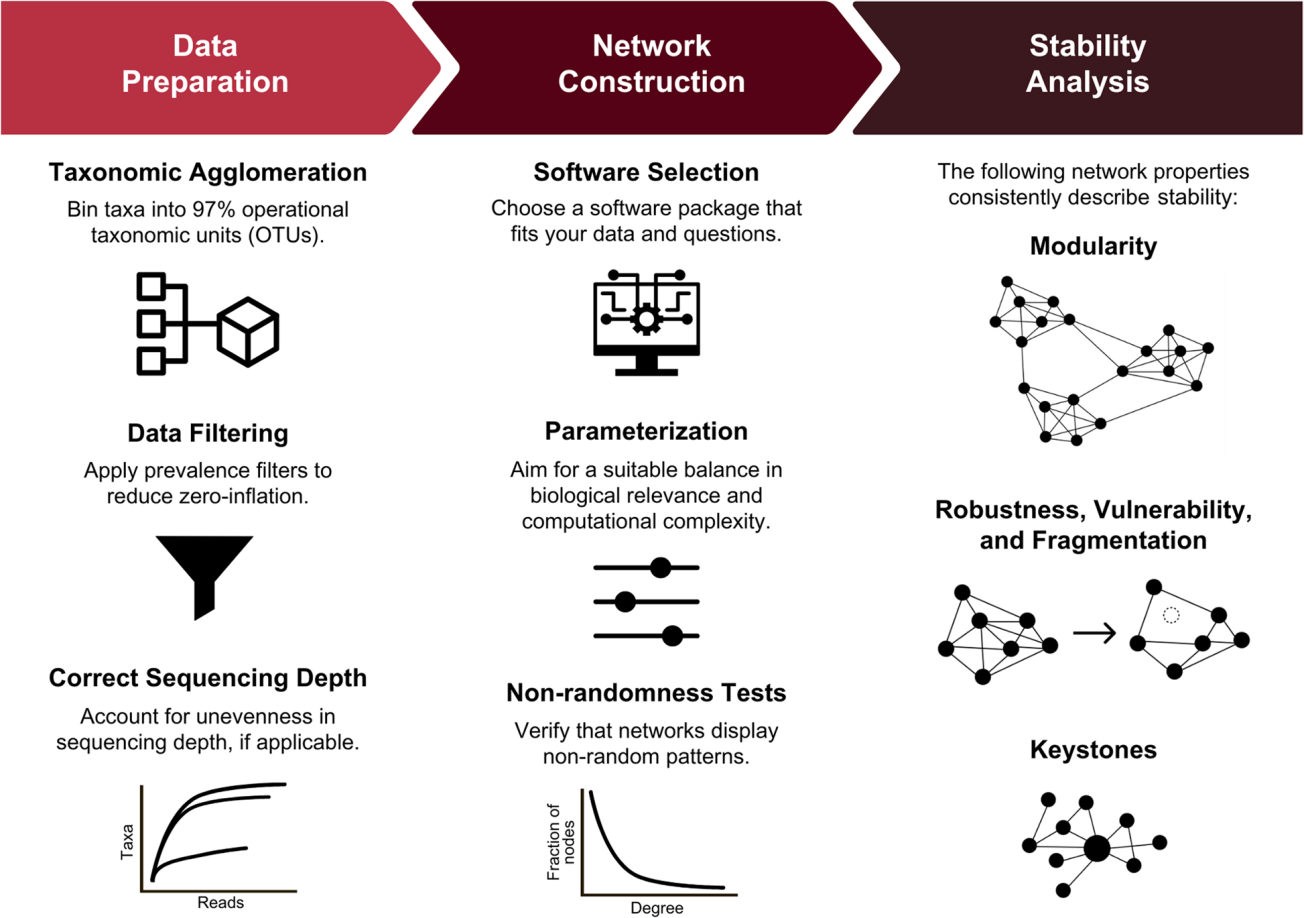
Example workflow for microbial network stability analyses. To prepare amplicon sequencing data for network assembly, data are commonly center-log ratio transformed, clustered into 97% operational taxonomic units (OTUs; or kept at 100% amplicon sequence variants, ASVs), prevalence filtered, and controlled for uneven sequencing and sampling bias (via rarefaction or other normalization). General data preparation steps apply to other data types as well. To construct networks, researchers should spend time comparing the strengths and weaknesses of various co-occurrence network software and then fine-tune their parameters. After networks are built, we suggest that they be tested for nonrandomness. Analyses should center on network modularity, robustness, vulnerability, fragmentation, and the presence of keystone nodes, as these have the most consistent interpretations with network stability

The following comprises a user’s guide for generating co-occurrence networks and implementing downstream analyses such as the assessment of stability (Fig. 1). From raw DNA sequencing data to network stability analysis, we outline considerations for data curation and software selection, as well as collate network topological metrics and network properties used to study stability. We highlight areas of congruence and incongruence on which metrics and properties indicate stability, review the remaining challenges in this area of study, and share suggestions for future work.

## Stage 1: Data preparation

Networks can be used to ask a range of biological and ecological questions, offering a window, for example, into the predicted importance of certain taxa in an ecosystem [12], potential drivers of community assembly [17], or the robustness of the entire system across disturbance gradients [18, 19]. To ask such questions, the data must first be curated to ensure that the inferred network is biologically and ecologically relevant to the study system and questions. This involves meeting the statistical expectations for inferring the network, minimizing the likelihood of spurious connections, and constructing networks in a manner that ensures reliable comparisons between them.

### Taxonomic agglomeration

Many microbiome network studies cluster sequences into operational taxonomic units (OTUs) at 97% sequence similarity (e.g., [18–20]), or amplicon sequence variants (ASVs) delineated by single-nucleotide differences [21–25], while some examine networks among higher taxonomic groupings such as genera or classes [26, 27]. The level at which microbial sequencing reads are binned and the taxonomic grouping affects what each node represents and what is indicated by edges between nodes. For example, cross-domain networking among classes of bacteria and fungi may represent higher-level ecological and biological interactions, whereas networks built from barcode loci ASVs may indicate distinct patterns among more closely related microbes. Researchers should consider what level of taxonomic resolution and sequence agglomeration is appropriate for their questions of interest [28–30]. It is also possible to cluster ASVs into OTUs while maintaining ASV seed sequences, which enables conversions between groupings as desired (as in [31]).

Binning taxa into 97% similarity OTUs or higher taxonomic groupings instead of ASVs also brings the benefit of reduction in dataset size and zero inflation (discussed in the next section). Feasible richness levels for network construction generally range in the order of hundreds to thousands of taxa (e.g., [24, 32]). The larger the dataset, in terms of taxa and/or samples, the more computational resources and time will be needed to build a network. Certain software have workarounds for batching out iterations or using lossy processes to speed up runtimes [33], though generally the pairwise nature of co-occurrence calculations makes network construction less amenable to parallelization.

### Data filtering

Microbiome data are often zero-inflated, which can cause erroneous predictions and lowered precision in correlation-based network methods, such as Spearman’s and Pearson’s correlations, *SparCC*, and the maximal information coefficient (MIC) [34]. Previous recommendations include taxa filtering to at least a 20% prevalence threshold to ensure that the interactions represented in the network are most likely to be biologically real and meaningful, though these cutoffs remain arbitrary and a subject of debate [35, 36], ranging anywhere from 10% [19, 21] to > 60% [37]. Depending on the question, more stringent filters may be appropriate, for example, network comparisons made within-host on human skin and lung microbiomes discarded taxa present in less than 33% of samples [13], while a comparison of soil microbiomes across different environments applied a 10% prevalence filter [19]. Data may also be pre-filtered to exclude pairings that cannot be reliably tested due to zero inflation [36], as a high incidence of zeroes not only complicates the detection of negative associations but can also artificially inflate positive correlations [35]. These filtering steps inevitably cull members of the rare biosphere, which has been touted in various systems for its ecological importance [38]. The spectrum of prevalence filtering thresholds from low to high generally represents a trade-off between inclusivity and accuracy. Data filtering will also reduce the dataset and subsequent computational burden of network construction. Here, as elsewhere in this guide, researchers are urged to consider these trade-offs in deciding what parameters make the most sense for their questions and systems.

### Sequencing or sampling bias

Rarefaction, the process of randomly subsampling to a set number of sequencing reads across samples, is commonly used in microbial network studies to address uneven sequencing depth [22, 24, 39]. There is strong debate on the appropriateness of rarefaction with regard to diversity analyses [40, 41], but the effects of rarefying in networks vary by data association algorithms used during network construction [42]. A comparison of correlation-based methods found that rarefying caused tools like *CoNet* and Spearman’s and Pearson’s correlations to suffer a decrease in precision, but that others such as *SparCC*, Bray–Curtis dissimilarity, and MIC could still reliably infer interactions [34]. The robustness of other network inference methods to rarefaction, such as graphical probabilistic models, has yet to be benchmarked.

To address sampling bias, sampling intensity may be held constant across treatments. For example, researchers may standardize samples to the lowest common replicate number across plots or individuals or use frequency distributions of reads by taxon or sample to maintain a standard proportion of the sampling effort [24]. As with prevalence filtering, these normalization methods will likely remove rare taxa.

### Compositional data bias

Microbiome data are compositional, in that counts represent proportions of taxa relative to the total number of sequencing reads in a sample, and not absolute abundances [43]. Observations in microbiome data are thus not independent, which breaks the assumptions of traditional correlation analysis and can result in a network with many false-positive signals [44, 45]. A common solution is to use the center-log ratio transformation to remove dependencies between proportions [45], either applied to the entire data table (as in *SPIEC-EASI*, a graphical method [33]) or to pairs of taxa (as in *SparCC*, a correlation method [44]). Alternatively, software using Dirichlet multinomial models aims to directly account for compositional data [46, 47].

### Inter-kingdom data

Specific data pre-processing steps are needed to create networks involving multiple domains of life, such as bacteria, archaea, fungi, and other microeukaryotes [13, 48–52], as their compositions are specific to each dataset and not the concatenated whole [53]. The *SPIEC-EASI* package automatically accommodates inter-kingdom data by independently transforming datasets with the center-log ratio transformation, which satisfies the equations to generate the inverse covariance matrix [13, 33]. Datasets used to make correlation-based networks should also be transformed independently before concatenation to avoid introducing bias and spurious edges [53].

## Stage 2: Network construction

### Software selection

At the time of writing, a broad suite of software is available for network generation, with each option offering different underlying models suitable for different needs [54]. Correlation-based methods are commonly used to infer links in microbial network construction [21, 24] but can be prone to issues involving compositionality and arbitrary significance cutoffs [54]. To promote statistical integrity in a correlation-based network, users may involve null models [55] or random matrix theory-imposed correlation cutoffs to separate organized information from noise [56]. The package *CoNet* employs an ensemble approach to reduce false positives, preserving only those edges supported across multiple correlation, similarity, or dissimilarity methods [57]. When using correlation-based methods, a false discovery rate should be used to correct for multiple comparisons (e.g., [58]).

Network software using probabilistic graphical models have also emerged to mediate several of the aforementioned challenges, along with the issue of indirect edges, or edges that arise due to shared responses to other taxa instead of a direct interaction. Graphical methods infer edges based on conditional dependencies, where an association is drawn between two nodes when there is a linear relationship between them, given all other nodes in the network [33], and an edge will not be drawn if that relationship can be explained by external taxa. Calculating networks in this manner often requires additional computational power [54]. One well-known algorithm in this family, *SPIEC-EASI*, was also designed to address the high dimensionality of microbiome data, where samples are often far outnumbered by taxa, which can otherwise result in overfitting [33].

Indirect edges may also result from common responses to environmental effects, such as pH, water availability, or mineral levels [42, 59]. Some packages are able to accommodate environmental data [57, 60], while other studies include environmental variables in their correlation analyses, either to observe how the environment structures co-occurrence patterns or to cancel them out [61–63]. Other suggestions to account for environmental effects include generating separate networks in instances where environmental data are expected to vary, for example, across water depths, as indirect edges should be less prevalent within a given environment [64]. It may also be possible to discern certain indirect edges resulting from geographic or environmental variation using phylogenetic distances to assess whether inferred edges are consistent with habitat filtering, dispersal limitation, or biotic interactions [65].

For an in-depth review on contemporary co-occurrence network software and their advantages and pitfalls, including packages suitable for metagenomic data, see [54]. Overall, difficulties remain across the gamut of network software in accounting for computational complexity and zero-inflated data [34, 54].

### Software parameterization

The parameters of a set of successfully constructed networks should have been fine-tuned so that the networks achieve a suitable balance in interpretability, biological relevance, and computational complexity, and are comparable among treatments. This may include choosing between Spearman’s or Pearson’s correlations, defining cutoffs for what is deemed a significant interaction, regulating network sparsity, or setting how many iterations of the calculation to perform [33, 56]. Different datasets will likely call for different parameters, but the selected values should always be biologically justifiable. For example, microbiome studies often favor Spearman’s correlations over Pearson’s, as the latter assumes normally distributed data with linear relationships, to which microbial data may not conform [55], and Pearson’s correlations may be less sensitive to detecting negative associations [36]. Networks resulting from the same type of model can also display a great deal of variation due to the freedom of choice inherent in parameterization [35]; thus, if different networks are to be compared, they should be constructed using identical parameters.

### Nonrandomness in networks

Finally, it is useful to confirm that the resulting networks differ from random networks, which have Poisson degree distributions [66, 67]. Networks are sometimes considered non-random if their topology differs from a distribution of topological values derived from random networks with the same node and edge numbers [56]. Often, studies have called networks non-random if they follow a power-law degree distribution (i.e., they are scale-free) and are considered small-world, or formed of more tightly knit clusters connected by relatively short paths [68]. However, nonrandom networks need only be non-Poisson and can take many different forms. Biological networks may also organize themselves similarly to random networks [69, 70], but are generally expected to be non-random [68]. Confirmatory studies with empirical and synthetic datasets are warranted to better define statistical expectations for non-random network structure in microbiomes especially, as these may differ from the macro-organismal, gene, and protein networks from which many of these tests are derived [68, 71].

## Stage 3: Network topology implies properties related to stability

**Fig. 2:**
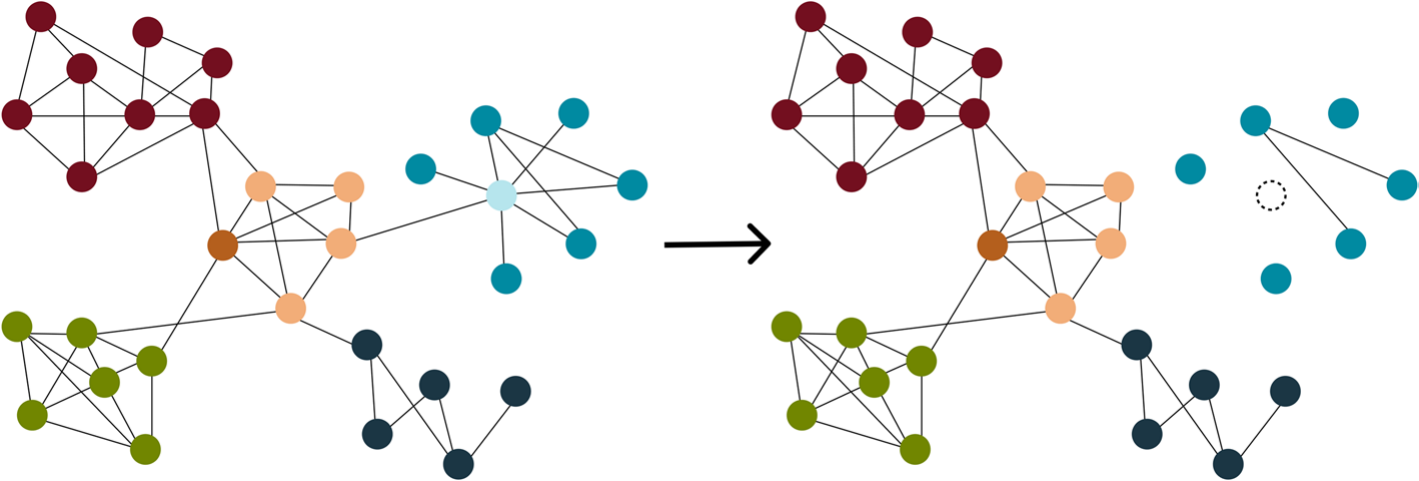
Visualization of network metrics commonly used to assess stability. The hypothetical network is partitioned into five modules, each distinguished by color. Nodes are much more connected within-module than among modules. The dark red module has a higher incidence of edges, indicating a relatively higher connectivity and linkage density compared to the navy blue module. Modules in dark red, orange, and green have higher clustering coefficients than modules in turquoise and navy blue, as they cluster into multiple “triangles.” The node in dark orange is an example keystone, as it has a high degree (connections to other nodes) and high betweenness centrality (it frequently lies on the shortest path between all pairs of nodes) and low relative prevalence (not shown). The node in light blue has relatively high vulnerability, in that its removal has a large effect on network efficiency or how traversable the network is. The loss of the vulnerable light blue node also leads to fragmentation of the turquoise module and a decrease in overall network robustness

After networks are constructed, their topology and other properties can be used to assess factors such as stability, though their interpretations can be clouded by different, even contradictory explanations for what values imply a stable network. The following sections describe various network metrics (Fig. 2) and how microbiome studies have positioned them in the context of stability (Table 1).

**Table 1:** Network topological metrics and network properties used in microbial network stability studies, with references. The majority of the listed metrics can be calculated in the igraph package [72]. For calculating network robustness, we suggest the packages brainGraph [73] or NetSwan [https://cran.r-project.org/web/packages/NetSwan/index.html]

Metric	Definition	High value = stable	Low value = stable
Average path length	The average shortest path (number of edges) between all pairs of nodes	[23, 26]	[39]
Betweenness centrality	Of a node, frequently occurring on the shortest path between all pairs of nodes	[23]	[20, 24, 74]
Connectivity	A measurement of how many connections are formed between nodes in a network	[18, 26]	[20, 25, 39, 58, 75]
Clustering coefficient/transitivity	The degree to which nodes in a network cluster together	[26, 39, 71, 76]	[20, 58]
Degree	The number of edges connected to a node	[76, 77]	[20, 58, 75]
Linkage density	The ratio of realized network edges to the total possible number of edges	[18, 24]	[58, 75]
Modularity	The partitioning within network communities into distinct and highly connected subcommunities	[18–20, 23–25, 39, 58, 77–79]	[75]
Network size	The total numbers of nodes in a network	[24, 39, 76]	
Vulnerability	The maximum impact of a node’s removal on network efficiency		[39, 77]
Cohesion	A measurement of the abundance-weighted positive and negative correlations in a network, standardized by a null model. Or more simply, the ratio of negative to positive edges	[18, 19, 23, 24, 77, 79]	[20, 21, 26, 39, 48, 58, 75]
Complexity	Various definitions (see Table 2)	[18, 19, 21, 26, 39, 77]	[25, 58, 75]
Fragmentation	The number of disconnected subgraphs network divided by the total number of nodes, upon stepwise node removal	[22, 24]	
Inter-kingdom associations	Networks involving interactions between microorganisms from different kingdoms (e.g., fungi with bacteria)	[13, 23]	[48]
Keystone taxa	A taxon with disproportionate importance on ecosystem function and stability in relation to its biomass. In networks, nodes with high betweenness centrality and degree or high connectivity within and among modules	[24, 39, 39, 76, 77, 79]	
Robustness	Networks able to resist collapse upon targeted node removal	[13, 22, 23, 25, 39, 48, 58, 75–77]	

### Network topological metrics and properties

#### Modularity

Modularity is a measurement of network partitioning into distinct and highly connected subcommunities or modules. Members of modules are thought to associate with one another due to shared functions, strategies, or environmental preferences [19, 61] and are only weakly connected to nodes in other modules. For example, in plant-pollinator networks, modules circumscribe coevolutionary units of taxonomically related species with convergent traits [80]. In microbiomes, modules may imply niche partitioning [64] and have been used to study habitat preferences [81]. Modules are desirable in terms of network stability because local disturbances are more likely to be contained within the module and not propagate. This idea of compartmentalization imparting stability is echoed in food web theory [82–85]. Other than one study that found stable networks to possess fewer modules [75], the literature is in strong agreement that high modularity indicates increased stability [18–20, 23–25, 39, 58, 77–79], though all of these studies were system-specific, with the majority focused on soil.

Modularity is considered a component of network complexity [18, 77, 77] and is sometimes itself a criterion for measuring stability [19, 58, 79]. There are many ways to delineate modules, and community detection remains an active area of research in network science [86, 87]. Commonly used algorithms in microbiome analysis include fast greedy clustering [88], random walks [88], and simulated annealing [89], ordered approximately by speed (high to low) and potential precision (low to high). See [11] and [90] for more in-depth comparisons of modularity algorithms in microbiome networks.

#### Betweenness centrality

Centrality is a metric that positions the contributions of individual nodes to network structure. Along with degree centrality (often reported simply as degree, see below), betweenness centrality is one of the most commonly used metrics in microbiome network studies. A node has high betweenness centrality if it frequently occurs on the shortest path between all pairs of nodes. Betweenness centrality has been used to computationally identify putative microbial keystone taxa [13, 91]. Nodes with high betweenness centrality have been termed “gatekeepers,” as their loss disproportionately affects the fragmentation of the network [24, 74]. These nodes may serve important roles in bridging network modules or distinct subcommunities [52].

Betweenness centrality can also be assessed as a global network property, for example, as the average or maximum betweenness centrality across all nodes [20, 39]. Low global betweenness centrality is often associated with higher stability [20, 24], although one study comparing networks across anthropogenic and natural disturbances found the opposite [23]. Thus, the relationship between betweenness centrality and network stability tends to be negative but is yet unresolved.

#### Connectivity

Network connectivity refers to the degree of connections formed between nodes in a network. Although this is a specific metric that can be calculated for individual vertices [72], studies often use this term while referring to other metrics such as degree (the number of connections to a node), linkage density (the proportion of realized links relative to the total possible number of links), or the total number of links (edge number). These metrics are described in further detail below. A more connected network may have a higher incidence of generalist species with broader niche preferences and potentially broader interaction breadths; for example, in plant-animal interaction networks, generalists are more densely linked [92].

To date, studies have not reached a consensus on the role of connectivity in stability. Some have postulated that high connectivity is stabilizing because it offers redundancy, where a higher density of connections between nodes and network compartments compensates for the loss of certain edges in the event of disturbance [26]. Connectivity has been described as increasing stability because a higher density of links may increase network complexity, which has been tied to stability [18]. Others argue that a high level of connectivity makes a system less stable since it is more vulnerable to cascade effects, where disturbances may propagate more easily through the network [39]. Connectivity may also reduce stability more indirectly, by decreasing the specificity of links (i.e., a highly connected node exhibits more “generalist” characteristics), which in turn reduces modularity [25, 92].

#### Degree

Node degree, or degree centrality, measures the number of edges connected to a node and is either taken as is or normalized by the total number of connections in the network. When the average node degree is used as a proxy for connectivity, networks with low degrees are usually associated with stability [20, 58, 75]. Degree can also be used to identify important nodes, in which a high degree indicates a hub and potential keystone taxon, and a higher incidence of keystones may indicate increased global network stability [76, 77]. High average degree may also indicate high network complexity [25], but various interpretations of stability with complexity abound (see “Complexity” section).

#### Network size

The size of a network is measured by the total number of nodes. A large network may increase its resistance to perturbation [76], and in general, this metric is seen in association with stable networks [24, 39]. However, the level of network stability is predetermined by other metrics such as robustness (i.e., network size itself does not suggest stability).

#### Clustering coefficient or transitivity

Clustering coefficient, also known as transitivity, measures the degree to which nodes’ neighbors are connected with one another, which indicates the communicability of the network. In microbiomes, higher clustering coefficients have been speculated to suggest cross-feeding relationships [92], especially when predicted associations are positive [62]. A high clustering coefficient coupled with low path length suggests a small-world structured network [77], which is efficient but potentially less stable due to vulnerability to node loss [39]. Indeed, increased efficiency may imply that these networks are less error-tolerant, making them more vulnerable to extinction events [68, 93]. High clustering coefficients have been associated with increasing levels of network degradation and the reduction of modularity, which by extension reduces stability [58].

High clustering coefficients also suggest a more compact/aggregated co-occurrence pattern, which has been seen as stabilizing due to more efficient resource transfer [76]. Similarly, [26] found compactness to be a feature of their stable networks.

#### Average path length

The average path length is another measure of network efficiency and is measured as the average shortest path between all pairs of nodes. The interpretation of path length with stability depends on whether studies position efficiency as enhancing or diminishing stability. A smaller average path length indicates a more compact network, which has been associated with more efficient resource sharing and thus higher stability [26], as well as the opposite, with increased vulnerability to node losses [39]. Higher average path lengths have also been associated with stable sites compared to disturbed sites [23].

#### Linkage density

Linkage density, also known as graph density or network density, is the ratio of realized edges to the total possible number of edges. Higher linkage density may increase the complexity of a network, increasing its stability [18]. However, May’s 1972 theorem indicates the opposite [83], where systems become less stable as complexity increases; a microbiome network study across a permafrost degradation gradient supports this [58]. Both schools of thought originate from food web theory [9], and both are found in microbiome networks, where lower [75] and higher [24] levels of density are said to describe stable networks. When linkage density is weighted by association strength, it is referred to as connectance (not to be confused with connectivity), and increased connectance has been associated with increased stability [18, 39].

#### Vulnerability

Vulnerability is calculated as how strongly a node contributes to the global efficiency of the network, where a node’s vulnerability is the loss in network efficiency when the node and all of its edges are removed [16]. The maximum node vulnerability represents the vulnerability of the entire network. Low vulnerability indicates a more stable network [39, 77]. Given that vulnerability is calculated on the basis of network efficiency (i.e., the compactness of the network, see “Average path length” section), this viewpoint also assumes a positive relationship between efficiency and stability.

#### Complexity

The complexity-stability debate stems from decades earlier in the food web literature, where MacArthur [94] purported that an increased number of species and links increased stability, while May [83] found the opposite. Beyond degree and connectivity, network complexity has been described in many ways over the years, from combinations of topological metrics [18, 25, 39, 75, 77] to the diversity in interaction strength [18, 77] or interaction sign [19, 23, 77, 79].

As a result, there is no single, accepted way to define a complex network (Table 2), but other than a few studies that found stable networks to be less complex [25, 58, 75], the literature has more or less converged on the viewpoint that increased complexity confers stability in microbiome networks [18, 19, 21, 26, 39, 77], with support in macroecological food webs [84, 95–97]. See [9] for an in-depth review of complexity and stability in ecological networks.

**Table 2:** Network metrics and properties used to indicate complexity

Metric	References
Cohesion	[19, 77]
Connectance	[18, 39]
Connectivity	[18, 39, 75]
Degree	[21, 25]
Evenness of association strength	[18]
Linkage density	[18, 58]
Modularity	[18, 25]
Network size	[25, 39, 75]
Number of keystones	[39]
Ratio of edges to nodes	[26]

#### Robustness

Robust networks are those that are able to resist rapid collapse in the face of disturbance. It is one of the few metrics with strong agreement in the literature, where greater network robustness equates to greater stability [13, 22, 23, 25, 39, 48, 58, 75–77].

Disturbances are imposed computationally via targeted node removal (usually in order of decreasing node importance, or randomly), and the remaining largest structure in the network is divided by its starting size after each node removal [98]. Larger proportions are considered more robust, as the network has maintained a greater extent of its connections despite the loss of highly connected nodes. This method is likened to the idea of extinction cascades, where the loss of one species leads to the loss of others dependent on it [97].

Robustness is alternatively measured by “natural connectivity,” a method also involving targeted node removal [99]. Both forms of robustness are generally plotted as a curve tracking values of network “wholeness” (e.g., the fractional size of the largest remaining network component; *y*-axis) over the number of nodes removed, ranging from zero to the total number of nodes in the network (*x*-axis). A larger area under the curve [13, 48] or lower absolute value of the slope [23] indicates greater robustness. To derive a single robustness value for a network, some studies have used the number of remaining connections after removing a certain proportion of nodes, for example, 50% of nodes [77] or five module hubs [22, 39].

#### Fragmentation

Fragmentation is calculated by dividing the number of disconnected subgraphs in a network (i.e., a network whose nodes and edges are subsets of a larger graph) by the total number of nodes following stepwise node removal [100]. Lower fragmentation values are indicative of greater stability [22, 24]. The loss of potential “gatekeeper” nodes with high betweenness centrality can lead to greater fragmentation of networks [100].

#### Cohesion (negative-to-positive interaction ratio)

Though many of the aforementioned metrics rely on nonnegative edges to calculate, positive and negative edge weights can be incorporated when assessing cohesion. Cohesion measures the abundance-weighted positive and negative correlations in a network, standardized by a null model [101], though sometimes studies will calculate the ratio of negative-to-positive interactions alone to assess stability [23, 24]. A higher ratio of predicted negative interactions is thought to impart stability to a network because such a network is better able to contain the negative effects of disturbances [102]. Positive interactions can lead to strong positive feedback loops, in which the loss of one species leads to the mutual downfall of all others with which it is linked. Approximately half of surveyed studies using this metric found a higher rate of negative interactions to be indicative of stable networks [18, 19, 23, 24, 77, 79]. This is also consistent with the stress-gradient hypothesis [103], where positive interactions increase in adverse conditions thought to destabilize communities. In microbiomes, this effect may occur via a proliferation of stress-tolerant species and/or facilitative associations [19].

Other studies find a greater incidence of positive interactions to either underpin [21, 26, 39, 48, 75] or describe [20, 58] more stable networks. Under this paradigm, greater levels of cooperation may increase the overall efficiency of resource transfer and imply stable coexistence across species [21, 48].

#### Keystone taxa

Keystone taxa were those originally hypothesized by Paine [104] to be disproportionately important for ecosystem function and stability in relation to their prevalence or biomass. Unlike in macro-organismal systems, where a potential keystone organism may be physically removed or naturally absent, the keystone phenomenon is challenging to directly apply and observe in microbial systems due to their often diverse and ephemeral nature. Instead, networks have been adopted to identify potential keystone taxa, or highly important nodes, which are then removed computationally as a test of their importance for maintaining network structure. To date, the literature is in agreement that keystones have a positive relationship with stability [24, 26, 39, 75, 77, 79].

Keystones are thought to be important for network stability because they uphold important structures in the network. Various methods exist to identify putative keystones. Some are based on network topology metrics, such as using degree to pinpoint hubs, while others use a combination of degree and betweenness centrality [13, 91]. These nodes are then further filtered by relative abundance to maintain low-abundance candidates as a parallel to macro-organismal keystone species properties. Another method ascribes topological roles to nodes based on within- and among-module connectivity [80, 89]. Keystones identified with this method are not necessarily screened for low abundance.

As with some other ecological “rules” primarily derived from the study of macro-organisms [105], it may be that the traditional definition of a keystone species does not directly apply to microorganisms, or at least warrants flexibility [106]. Microbial metacommunities do not seem to be defined by habitable patches but rather stepping stones that facilitate dispersal across distance and disjunct suitable habitats [107], and as such, microbes may not be restricted to the contexts (i.e., hosts, habitats, or environments) in which keystone-like behavior is likely to be observed [106]. Experimental validation is necessary to determine the plausibility of microbial keystones [12, 14], as network-based simulations of putative keystone removal rely on the assumption that network edges represent biotic associations, which may not be the case in co-occurrence networks [35].

#### Inter-kingdom interactions

When inter-kingdom interactions including both prokaryotic and eukaryotic microbes are included in co-occurrence networks, it is often found that stability increases, generally as a result of the addition of fungi [13, 23], which act as connectors between network modules [79]. Multiple hypotheses may explain this phenomenon: fungi may produce metabolites that bacteria may exploit when nutrients are limited [108], provide physical space for bacterial colonization and dispersal [109], or support bacterial resistance to hydric stress [23]. One study of host evolution in wild and domesticated rice found a different effect, where the addition of fungi to bacterial networks decreased network robustness but increased transitivity (i.e., clustering coefficient) and modularity [48]. In this study, stability was indicated by network robustness, and the reduction of stability in the presence of fungi was attributed to the loss of fungal-bacterial modules [48]. More integrative microbiome research is needed, as many studies focus on bacteria alone [18, 24, 39, 76, 77, 79], but the addition of multiple guilds and/or kingdoms may change stability assessments [52].

## Remaining challenges

One of the largest shortcomings of contemporary network analyses is the inability to make robust statistical comparisons between networks. Networks require a sufficient sample size to reliably infer interactions [33], but this often means every sample is put towards meeting a suitable sample-to-feature ratio and not towards generating replicate networks that would be needed to create distributions of topological metrics or other properties. Thus, we often cannot say that one network is significantly more modular than another, for example, because only one modularity value can be calculated per network. Some studies have used subnetworks corresponding to individual plots from a single empirical network to make statistical comparisons across sites [18]. Researchers must take care to not pseudoreplicate if taking this approach, ensuring that spatial autocorrelation  and proper replication  are considered with respect to their questions.

When interpreting co-occurrence networks, it is important to consider the biological or ecological relevance of a given pattern, or the degree to which such relevance is attainable [64]. Data preprocessing can help to reduce spurious and indirect edges (see above), but even then, networks have limitations in representing biotic signals, as correlations may not capture asymmetric, directional associations such as trophic interactions [110]. Network structure may also be driven by various factors, including environmental filtering, dispersal limitation, stochastic processes, or biotic interaction, and the interpretations of a given network metric or property should consider the role of these factors. For example, when most links are driven by abiotic variation, calculating network robustness via simulated species extinction may not be biologically relevant. However, it remains difficult to determine which edges and network structures are governed by specific ecological processes (but see [65]); thus, future network interpretations will likely be constrained by this caveat.

Co-occurrence networks should be used to generate, and not validate hypotheses [35], especially those that assume biotic interactivity (e.g., ratios of cooperation to competition). Regardless, networks have proven utility in studying ecological theory (e.g., [106]), allow complex, species-rich microbial communities to be more interpretable, and will likely remain useful tools to guide the direction of resources in more intensive pursuits, such as co-culturing or whole genome sequencing.

The development of comprehensive tools for network generation will help ensure that resulting networks represent “interactomes” to the greatest possible extent [61]. Current tools offer some, or most, but not all of the functionality needed to do this, including the ability to handle rare species, the compositionality and high dimensionality of microbiome data, indirect edges, environmental effects, multiple domains of life, and large amounts of taxa, while minimizing the use of arbitrary thresholds. Added functionality will require more computational power, so options to compute networks in batches or in a lossy manner may be necessary. As methods evolve and converge, there will hopefully be less variability among software in what edges are inferred [34].

## Conclusions

Research into microbiome networks is challenged by a forked path of decision-making and the existence of multiple plausible interpretations and few accepted standards. For example, of the network properties described in this review, it is generally agreed that stable networks are those partitioned into many distinct modules (high modularity, [18–20, 23, 25, 39, 48, 58, 75–77]) that are resilient in the face of targeted node removal (high robustness [13, 22, 23, 25, 39, 48, 58, 75–77]), have low vulnerability [39, 77] and low fragmentation [22, 24], and are held together by keystone taxa [24, 26, 39, 75–77, 79]. Although certain network metrics correlate with these properties (e.g., degree with robustness or keystone designation), a suite of topological metrics should be considered when describing stable networks.

One potential reason for the lack of convergence between network metrics and their interpretations across studies is that the majority of studies looked at single sample types (mostly soil; [18–21, 23, 25, 26, 39, 75, 78, 79]) and single domains of life (mostly bacteria; [18, 24, 39, 76, 77, 79]). A strong need for the field is to assess networks representing the diversity of ecosystems in situ to differentiate system-specific stability quirks from universal traits.

With further confirmatory experiments, benchmarking work on microbiome data, and computationally scalable tools able to handle the multidimensionality of the data [42, 54], co-occurrence networks can transform from being an incredibly informative tool to one capable of robust hypothesis generation and testing.

## Data Availability

No datasets were generated or analysed during the current study.
